# Tai Chi Improves Coronary Heart Disease Risk by Inactivating MAPK/ERK Pathway through Serum miR-126

**DOI:** 10.1155/2020/4565438

**Published:** 2020-04-25

**Authors:** Guangwei Zhang, Shuli Wang, Yan Gu, Ling Song, Shui Yu, Xiaoxing Feng

**Affiliations:** Department of Cardiovascular, The First Hospital of Jilin University, Changchun 130021, China

## Abstract

**Background:**

Tai Chi is effective in preventing heart disease (CHD) risk, but the molecular mechanism remains unclear. Mitogen-activated protein kinase (MAPK) signaling plays a critical role in the pathogenesis of CHD and can be activated by miR-126. Tai Chi may exert its protective function through the miR-126-modulated MAPK pathway.

**Methods:**

The CHD patients after PCI were randomized into the CG group (CG) (n = 19, normal care) and Tai Chi group (TG) (Tai Chi intervention, n = 17). Epicardial adipose tissue volume (EATV) (one main adverse cardiovascular event of CHD), HR (heart rate), QoL (quality of life) scores, and balance performance were measured in the two groups. The body fat content, abdominal subcutaneous fat, and visceral fat were measured to reflect the improvement of adipose tissue dysfunction. The levels of miR-126 and MAPK-associated molecules were measured in peripheral blood leukocytes. Meanwhile, the effects of miR-126 silence and mimic on MAPK-associated molecules were also explored in cardiac cell H9C2.

**Results:**

After the 3-month intervention, Tai Chi reduced EATV and HR and increased QoL scores and balance performance, respectively (*P* < 0.05). The fat percentage, body fat mass, and BMI were also significantly reduced after Tai Chi intervention (*P* < 0.05). The levels of miR-126, MAPK, JNK, and ERK in the TG group were lower than those in the CG group (*P* < 0.05). The miR-126 levels had a strong relationship with the values of EATV, HR, and QoL scores (*P* < 0.05). miR-126 silence or mimic inactivated or activated MAPK-associated molecules in the cardiac cell lines.

**Conclusions:**

Tai Chi improved CHD risk by inactivating the MAPK/ERK pathway via serum miR-126.

## 1. Introduction

Coronary heart disease (CHD) is a disorder of cardiac function due to severe atherosclerotic stenosis or obstruction occurs in the coronary arteries, and thrombosis causes luminal obstruction, resulting in coronary insufficiency, myocardial ischemia, or infarction. The trend of cardiovascular risk factors has increased significantly, leading to a continuous increase in the number of CHD cases [1]. The overall mortality of CHD is still increasing too [2].

Percutaneous coronary intervention (PCI) is the main approach in the treatment of CHD [3]. PCI surgery can quickly restore coronary blood circulation, improve myocardial ischemia, and protect heart function [4]. Based on medical treatment, exercise-centered cardiac rehabilitation is also effective in the prevention of CHD progression [5]. With the development of PCI, cardiac rehabilitation has gradually extended to the rehabilitation of the CHD patients after PCI [6]. Heart rate (HR) >76 bpm is at a higher risk of major adverse cardiovascular in CHD patients after PCI [7], and quality of life (QoL) is often used to assess health status after PCI [8]. Obesity is closely associated with heart failure [9, 10] and adipose dysfunction [11–13]. Epicardial fat is visceral thoracic fat and known to be related to the presence of dyslipidemia and coronary arterial stenosis in the patients after PCI [14]. Epicardial adipose tissue volume (EATV) is an independent indicator of long-term main adverse cardiovascular events in CHD patients after PCI [15] and affected by adipose tissue dysfunction. HR variability is also influenced by epicardial fat [16]. HR and blood pressure (BP) are interacted [17]. The inhibition of sympathetic nerve activity of adipose tissue has been found to reduce HR and BP [16]. An animal test showed that oxygen breathing affects adipose through air bubble [18, 19]. Breath exercise can reduce HR [20] and BP [21] and increase QoL [22] and may have the protective function for the patients after PCI by affecting adipose.

Tai Chi is used as a nursing treatment or rehabilitation method to prevent CHD risk [23, 24]. During Tai Chi training, deep breathing and mental concentration will be required to achieve the harmony between body and mind [25]. Tai Chi intervention has been reported to control body weight, reduce HR and BP in the patients with heart failure [26], and promote emotional self-regulation [27]. It can be seen that Tai Chi program also affects the risk factors of CHD including hypertension, hyperlipidemia, hyperglycemia [28], obesity, and psychology and improves the QoL of patients [29, 30]. However, the related molecular mechanism of Tai Chi exercise remains unclear. Mitogen-activated protein kinase (MAPK) signaling plays a critical role in the pathogenesis of CHD [31, 32] and can be activated by miR-126 [33, 34]. miR-126 is an independent risk factor of CHD and affects many important gene expression [35, 36]. Tai Chi may exert its protective function via miR-126-modulated MAPK pathway, and related work was performed here. Meanwhile, we also explored the effects of Tai Chi on EATV, HR, BP, QoL, and adipose changes in patients after PCI.

## 2. Methods

### 2.1. Participants

Before the study, all the procedures were approved by the Ethics Committee of The First Hospital of Jilin University (approval no. 2015JLU23F). Social-demographic and clinical information on CHD patients were evaluated. From September 2015 to February 2016, the patients who were discharged from our hospital were selected according to the inclusion and exclusion criteria. After the baseline data collection, the random number table was assigned to the Tai Chi group (TG) and the control group (CG) according to the allocation concealment.

### 2.2. Inclusion Criteria

All patients underwent PCI, which is a method of improving coronary perfusion by puncturing a blood vessel, delivering a catheter to the opening of the coronary artery, and using special materials and techniques to dredge stenotic or occluded blood vessels. This study was limited to narrow stent implantation. The age was 45–75 years old and HR > 60 beats/min. All subjects lived in Changchun City. All patients were conscious, had normal thinking, and could communicate normally.

### 2.3. Exclusion Criteria

The patients who had arrhythmia, chronic respiratory diseases, severe liver and kidney disease, r severe cognitive dysfunction, mental illness, and or uncoordinated examination were excluded. The patients requested to withdraw their informed consent and terminated the Tai Chi exercise due to an emergency during the intervention.

### 2.4. Sample Size Calculation

The sample size was calculated by using the equation as follows: *n*_1_ = *n*_2_ = ((*Z*_*α*_+*Z*_*β*_) *σ*/*δ*)^2^ + *Z*_*α*_^2^/4. In the formula, *n*_*1*_ and *n*_*2*_ stand for the number of samples required for each group, σ is the standard deviation of the research index, and *δ* is the difference between the two mean values of the research indicators. *Z*_*α*_ and *Z*_*β*_ are *Z* values corresponding to the type I and II error probability *α* and *β*, respectively. The sample size (*n*_1_ = *n*_2_ = 13.6612 ≈ 14) was required for each group. Considering the 10% sample shedding rate, the total sample size was expected to be 31 cases.

### 2.5. China Questionnaire of Quality of Life in Patients with Cardiovascular Disease (CQQC) [37]

The CQQC scale is a cardiovascular disease-specific scale developed by the Chinese Society of Rehabilitation Medicine Cardiovascular Diseases Committee. The score range is 0–154. Cronbach's coefficient of the questionnaire is above 0.76. The scale has good reliability and validity for all patients with cardiovascular disease and patients with hypertension, heart failure, and CHD, and it is closely related to health survey summary (SF-36).

### 2.6. Evaluation of Physical Activity and Diet

Physical activity and diet will affect the patient's weight and heart function, and they were measured as follows. Physical activity scale was established according to four questions: usual frequency of physical exercise, taking part in sports, taking long walks, and swimming. The responses can be never (0 score), sometimes (1 score), and often (2 scores) for each item, respectively. Physical activity was divided into low, medium, and high degrees (scores of 0–2, 3–5, and 6–8, respectively). Daily diet intake was measured by using a food frequency questionnaire (FFQ) and the consumed food was converted to grams according to a previous report [38]. The data were collected from the two groups during the 3-month experiment.

### 2.7. Medical Treatment

Traditional Chinese medicine was used for all patients, including Danshen (20 mg/kg/day) [39] and Suxiao Jiuxin Pills (18 pills/day) [40]. The number of patients taking different medicines was compared.

### 2.8. Tai Chi Intervention

Tai Chi training was performed according to a previous report [41], and the subjects paid more attention to stretch their necks, shoulders, hips, knees, and ankles. Balance was exercised from a sitting position to stand on one leg, walk tandem, backward, and sideways, and turn around. The exercise should be performed for 90 min and start at 8 am each morning.

The breathing patterns of Tai Chi consisted of breath frequency, depth, breath/inhalation time ratio, and chest/abdominal breathing. In the abdominal breathing, during the process of breathing accompanied by the movement of the diaphragm, the abdomen was raised when inhaling, and the abdomen was depressed when exhaling. The abdominal breathing method using bilateral nasal sniffing was specifically used in the Tai Chi exercise. Before the formal intervention, the study was conducted to carry out clinical pre-experiment of hospitalized CHD patients after PCI and to improve the Tai Chi program through pre-experiment. The breathing pattern was breathing frequency 6 times/min, and exercise frequency was 3 times/d for 30 min/time. The QoL was assessed using CQQC scores.

The patients in the Tai Chi group received breathing training. Specific content included taking a comfortable posture, such as sitting, standing, and sitting-in, straightening the head and spine, closing your eyes, and relaxing body. Using a prolonged exhalation and or inhalation time slowed down the breathing rate. Tai Chi could be combined with the abdominal method to enhance the depth of breathing. The whole process was carried out using nasal suction, and the intensity of the exercise was not suitable for breathless exercise and no discomfort. When there was discomfort during the practice, the subjects could take a break and continued without any discomfort. The whole period of exercise was 3 months.

The following information was added in the 2.8 Tai Chi intervention section.

It is difficult to ensure the quality of Tai Chi performance although its beneficial effects on heart disease have been widely reported. The previous study showed that posturography may be an effective way to measure the quality of Tai Chi and the expertise of Tai Chi practitioners was potentially associated with health outcomes [42]. The posturography of CHD patients was measured by using the Computerized Assessment of Postural Stability (CAPS) (Vestibular Technologies, Cheyenne, WY, USA) balance testing [43]. The test was conducted in the afternoon after Tai Chi or normal physical exercise, which included a combination of the eyes-open, eyes-closed assay on firm or unstable surface. The CHD patients were demanded to stand on a foam cushion in the center of the platform for eyes open (20 s) and then eyes closed (20 s).

### 2.9. Control Group

In the control group, all CHD patients kept the usual lifestyle and an equal amount of physical activities as Tai Chi group, including walking, dancing, gardening, stretching, stationary biking, and leg strengthening. The physical activity was evaluated by using an activity monitoring and evaluation system (A-MES, Kumamoto, Japan) in both groups. Two A-MESs were attached to the body to measure the consecutive activity. The system consists of two 3D posture and acceleration sensors for chest, thigh, and data recorder. The data of physical activities included supine, sitting and standing position, and walking duration and were recorded during a day. The patients from both groups joined the health education lesson in different community centers. A balance test was also performed in the control group.

### 2.10. EATV Measurement

EATV was measured by using 64-slice spiral computed tomography in the CHD patients. The expert drew the boundary of the heart in the transverse, sagittal, and coronary views using the tools of the workstation. The cardiac volume was confirmed by indicating a pericardium wall between two lines. The volume was determined by using CT values. The normal EATV values were arbitrarily defined as 91.3 cm^3^ according to the mean values previously reported [44]. All steps were conducted by two experts and reached the same conclusion finally.

### 2.11. Measurement of Heart Rate and Blood Pressure

HR and BP are intimately related. HR and BP were measured by using a uniform electronic sphygmomanometer (OMRON, HEM-4011C, Kyoto, Japan). HR is the number of times the heart beats every minute while resting quietly. Systolic blood pressure (SBP) is the lateral pressure exerted by the blood in the ventricle on the vessel wall when the heart contracted. Diastolic blood pressure (DBP) is intra-arterial blood pressure exerted on the vessel wall in the end-diastolic phase, and rate-pressure-product (RPP) is the product of heart rate and systolic pressure. The normal value of RPP is <12000 mmHg times/min. The smaller the product, the more stable the patient's condition.

### 2.12. Body Shape Measurements

Body shape was recorded by measuring body weight, fat%, BMI (kg/m^2^), abdominal subcutaneous fat, abdominal visceral fat, and body fat content (torso, limbs, and head). Fat% was measured by using dual-energy X-ray to accurately measure fat percentage. The areas of subcutaneous abdominal fat (SAT) and visceral fat (VA) were measured by the CT scanning technique to measure the abdominal fat area of the subject with a scan voltage of 120 KVp and a current of 150 mA. During the test, the subject was asked to lie on the designated place, the arm was horizontally higher than the head, the position of the navel (between the fourth lumbar vertebra and the fifth lumbar vertebrae) was scanned for 2 seconds, and the image with a thickness of 5 mm was recorded for calculation and analysis. The visceral fat area was the fat in the peritoneal wall layer or the transverse fascia, excluding the spine and paraspinal muscles. The subcutaneous fat area of the abdomen was the superficial fat of the abdomen and back. The CT scans were performed by the same radiologist using a single-blind method.

### 2.13. Fat Content in Different Parts of the Body

Before the intervention and after 3-month intervention, the subjects were examined by using Dual-emission X-ray Absorptiometry (DEXA) to measure the fat content of various parts of the body, including body fat, head fat, leg fat, arm fat, and the areas of abdominal android and hip gynoid. Subjects were allowed to lie flat in the designated position before the test, and the measurement was started after lying down. The android area includes abdominal subcutaneous fat and visceral fat between the ribs and the pelvis. The gynoid area is an overlapping part of the thigh and torso. The test was completed by the same doctor. After the test, the doctor used Encre 9.20 software to analyze the data, and the correlation coefficient was measured twice.

### 2.14. Quantitative Real-Time PCR (qRT-PCR) Analysis

5 mL blood was obtained from each participant and peripheral blood leukocytes (PBLs) from 4 mL were taken using Histopaque-1077 by Sigma-Aldrich via density gradient. The serum was prepared from 1 mL blood via centrifugation at 2000×*g* for 10 min. Total RNA was obtained by using the RNA purification kit (Epientre, Chicago, IL, USA). 0.5 μg of RNA from each sample was reversely transcribed by using High-Capacity cDNA Reverse Transcription Kit (Applied Biosystems, Foster City, CA, USA). The following primers were used for real-time PCR and synthesized by Shanghai Sangon Biotechnology (Shanghai, China), miR-126 (forward primer: 5′-TGTGGCTGTTAGGCATGG-3′ and reverse primer: 5′-AAGACTCAGGCCCAGGC-3′), U6 snRNA (5′-CTCGCTTCGGCAGCACA-3′), MAPK (forward primer: 5′-ACGTTCTACCGGCAGGAGCT-3′) and reverse primer: 5′-AAGCAGCACACACAGAGCCA-3′), c-Jun NH2-terminal kinase (JNK) (forward primer: 5′-GGATATAGCTTTGAGAAACTCTTCC-3′ and reverse primer: 5′-TCTAACTGCTTGTCAGGGATCTT-3′), ERK (forward primer: 5′-GAGGTTGACCACGGTGGAAT-3' and reverse primer: 5′- TTTGGTTTCCCACGGCTTCT-3′), and *β*-actin (forward primer: 5′-tcctccctggagaagagcta-3′ and reverse primer: 5′-gcactgtgttggcatacagg-3′) as an internal control. Relative levels of miR-126 were measured from serum RNA, and other molecules were measured from PBLs RNA. The qRT-PCR reaction was carried out as follows: one cycle of 95°C for 60 s, followed by 40 cycles of 95°C for 5 s, 60°C for 15 s, and 1 cycle of 60°C for 45 s, and it was maintained at 4°C. Relative mRNA levels of target genes were normalized to the level of *β*-actin and calculated by using 2-ΔΔC T method.

### 2.15. Western Blot

PBLs were lysed using the Mammalian Cell Lysis kit from Sigma-Aldrich (St. Louis, MO, USA). The proteins were separated by 10% by 10% SDS-PAGE and transferred onto polyvinylidene fluoride (PVDF) membranes (Millipore, CA, USA). The membrane was blocked with 5% nonfat milk in TBST and then incubated with anti-MAPK (ab109225, 1 : 2000, Abcam), JNK (ab179461, 1 : 1000, Abcam), ERK (ab137619, 1 : 1000, Abcam), and *β*-actin antibodies (ab8227, 1 : 5000, Abcam) overnight at 4°C. The membrane was then incubated with secondary antibody Goat Anti-Rabbit IgG H&L (HRP) (ab205718, 1 : 3000) for one hour. Immunoreactivity bands were visualized using by ECL (Advansta, USA) and quantified using ImageJ (NIH, USA).

### 2.16. The Effects of miR-126 on MAPK Signaling Pathway

The cardiac muscle cell line of H9C2 was purchased from the Cell Bank of Shanghai CAS (Shanghai, China) and cultured in the RPMI 1640 supplemented with 0.5% penicillin/streptomycin and 10 mM HEPES in a 5% CO_2_ incubator at 37°C. miR-126 shRNA was synthesized by using miR-126 mimic 5′-ATTATTACTTTTGGTACGCG-3′ and or anti-miR-126 sequence: 5′-GCATTATTACTCACGGTACGA-3' (Sangon Biotech). The cells were transfected with miR-126 mimic or shRNA by using Lipofectamin2000 reagent (Invitrogen, USA). Thus, all cells were divided into three groups: CG, control group; IG, miR-126 silence group; and MG, miR-126 mimic group (*n* = 8 for each group).

### 2.17. Statistical Analysis

SPSS18.0 was used for statistical analysis. The change values of the indicators before and after the intervention were expressed by 95% confidence intervals between the two groups. The normal distribution data were analyzed by two independent samples and a nonparametric Mann–Whitney U rank-sum test. A repeated-measures ANOVA with two was used to compare the change from the baselines between the two groups. Pearson's correlation coefficient test was used to explore the relationship between the relative levels of miR-126 and EATV, HR, or QoL scores. *P* < 0.05 was considered statistically significant. The comparison of the count data between the two groups was performed using the *χ*^2^ test.

## 3. Results

### 3.1. Baseline Characteristics

The mean ages of the patients in this study were 61.00 ± 8.07 years old, 24 cases (66.67%) < 65 years old, 12 cases (33.33%) of 65–75 years old, and 34 cases (94.44%) males. A total of 36 patients were enrolled in the study, 19 patients in the TG group and 17 patients in the control group. At 3 months, 6 patients (16.67%) were lost to follow-up: 1 patient (5.26%) in the TG group was unable to complete the corresponding Tai Chi (the total exercise time < 150 min/w); five subjects were lost in the CG group (29.41%), of which 1 case could not be measured due to outing travel, and 4 cases were unable to be followed up. A total of 30 patients (83.33%) completed a 3-month study, of which 18 (97.74%) were in the TG group and 12 (70.59%) in the CG group.  Table 1 showed that the demographic data and clinical data of the two groups were insignificant (*P* > 0.05). Overweight criteria were often considered if BMI ≥ 23 [45] and most patients were overweight. The results indicated that the statistical difference for physical activity and daily diet intake between the two groups was insignificant (Table 1). Although Danshen and Suxiao Jiuxin have some effects on HR and BP [46, 47], the statistical difference for the number of patients taking medicine between the two groups was insignificant (Table 1, *P* > 0.05). The statistical difference for the physical activity was insignificant either, including supine, sitting and standing position, and walking duration (Table 1, *P* > 0.05).

### 3.2. Tai Chi Reduced EATV

As-treated analysis showed that the statistical difference for EATV between the two groups was insignificant (Table 2, *P* > 0.05). After the 3-month intervention, Tai Chi reduced EATV values significantly when compared with the CG group (Table 2, *P* < 0.05). In similar results, ITT showed that the statistical difference for EATV between the two groups was insignificant (Table 2, *P* > 0.05). After the 3-month intervention, Tai Chi reduced EATV when compared with the CG group (Table 2, *P* < 0.05).

### 3.3. Tai Chi Reduced HR and BP of CHD Patients

As-treated analysis showed that the statistical difference for HR and BP between the two groups was insignificant (Table 3, *P* > 0.05). After the 3-month intervention, Tai Chi reduced HR and SBP (Table 3, *P* < 0.05). In similar results, ITT showed that the statistical difference for HR and BP between the two groups was insignificant (Table 4, *P* > 0.05). After the 3-month intervention, Tai Chi reduced HR and SBP when compared with the CG group (Table 4, *P* < 0.05).

### 3.4. Tai Chi Reduced RPP

As-treated analysis showed that the statistical difference for RPP between the two groups was insignificant (Table 5, *P* > 0.05). After the 3-month intervention, Tai Chi reduced RPP when compared with the CG group (Table 5, *P* < 0.05). In the similar results, ITT showed that the statistical difference for RPP between the two groups was insignificant (Table 5, *P* > 0.05). After the 3-month intervention, Tai Chi reduced RPP when compared with the CG group (Table 5, *P* < 0.05). The results suggested that Tai Chi reduced RPP values.

### 3.5. Tai Chi Improved QoL Scores

As-treated analysis showed that the statistical difference for CQQC score between the two groups was insignificant (Table 6, *P* > 0.05). After the 3-month intervention, Tai Chi increased CQQC scores when compared with the CG group (Table 6, *P* < 0.01). In the similar results, ITT showed that the statistical difference for CQQC score between the two groups was insignificant (Table 6, *P* > 0.05). After 3-month intervention, Tai Chi increased CQQC scores when compared with the CG group (Table 6, *P* < 0.05). The results suggested that Tai Chi increased CQQC scores.

### 3.6. Basic Condition for Fat Contents

Before the intervention, there was no significant difference for age, body weight, fat%, nonfat weight, fat weight, the fat content of various parts of the body (arm, leg, gynoid area of the hip, and android area of the abdomen), and the areas of abdominal subcutaneous fat and visceral fat (*P* > 0.05). All these parameters would not affect interfere final results.

### 3.7. Comparison of Changes in Dietary Calories and Physical Activity

As shown in Table 7, the dietary caloric status of all subjects was not statistically different before and after the intervention (*P*=0.260). There was no statistical difference in physical activity between all subjects before and after the intervention (*P*=0.115), which ruled out the effects of dietary calories and physical activity on weight loss.

### 3.8. Tai Chi Intervention Reduced Weight Gain

Before the intervention, there was no significant difference in body weight between the two groups (Table 8, *P*=0.700). After the 3-month intervention, body weight decreased, and the statistical difference was significant (Table 8, *P* < 0.05). From the above results, the Tai Chi training program can effectively reduce the weight of obese patients.

### 3.9. Tai Chi Intervention Reduced Fat%

Before the intervention, there was no significant difference in fat% between the two groups (Table 8, *P*=0.437). After the 3-month intervention, fat% decreased, and the statistical difference was significant (Table 8, *P* < 0.05). From the above results, the Tai Chi training program can effectively reduce the fat percentage of CHD patients after PCI.

### 3.10. Tai Chi Intervention Reduced Fat Mass and Local Fat Mass

Before the intervention, there was no significant difference in fat weight and nonfat weight between the two groups (Table 8, *P* > 0.05). After the 3-month intervention, the fat weight decreased, and the statistical difference was significant (Table 8, *P* < 0.05) while the changes for nonfat weight were insignificant (Table 8, *P* > 0.05). Tai Chi training program effectively reduced the fat weight of obese patients, and there was no significant difference in the extent of decline. In similar cases, the statistical differences were insignificant for the contents of right leg fat, left leg fat, right arm fat, left arm fat, body fat, head fat, belly (android) fat, and hip (gynoid) fat between the two groups (Table 8, *P* > 0.05). After the 3-month intervention, the fat contents of different parts of patient body were significantly reduced except head fat (Table 8, *P* < 0.05). From the above results, the Tai Chi training program can effectively reduce fat contents of different parts of patient body.

### 3.11. Tai Chi Reduced the Areas of Visceral Fat and Subcutaneous Fat

Before the statistical analysis, the statistical difference for the areas of visceral fat and subcutaneous fat between the two groups was insignificant (Table 8, *P*=0.437). After the 3-month intervention, the areas of visceral fat and abdominal subcutaneous fat were reduced by 13.1% (68.9 ± 24.7 cm^2^ versus 59.9 ± 19.1 cm^2^, *P*=0.032) and 14.1% (248.4 ± 61.2 cm^2^ versus 213.4 ± 51.0 cm^2^, *P*=0.040) after the exercise intervention (Table 8). There were changes for CG from the above results, and the Tai Chi effectively reduced the areas of visceral fat and subcutaneous fat of CHD patients.

### 3.12. Tai Chi Improved the Balance Performance of the CHD Patients

The statistical difference for the average scores of balance stability was insignificant between the Tai Chi group and the control group before the intervention (Table 9, *P* > 0.05). After the 3-month intervention, the average scores of balance stability were significantly improved in the Tai Chi group when compared with the control group (Table 9, *P* < 0.05). The results suggest that Tai Chi improved the balance performance of CHD patients.

### 3.13. Tai Chi Reduced the Relative Level of miR-126 in the CHD Patients

**Figure 1:**
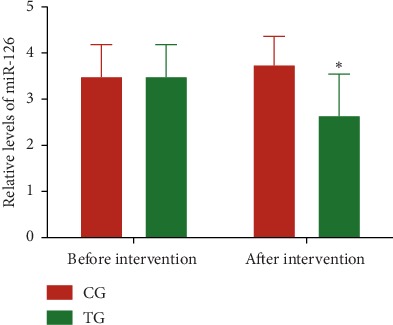
Relative levels of miR-126 in patients with coronary heart disease. TG, Tai Chi intervention group (*n* = 18); CG, common-care group (*n* = 12). The statistical difference was significant if *P* < 0.05.

Before the intervention, the statistical difference for miR-126 (Figure 1) was insignificant between the two groups (*P* > 0.05). After the 3-month intervention, the levels for miR-126 (Figure 1) in the TG group were lower than those in the CG group (*P* < 0.05). The results suggest that Tai Chi intervention reduces relative levels of miR-126 in CHD patients.

### 3.14. Tai Chi Reduced Relative mRNA Levels of MAPK, JNK, and ERK in the CHD Patients

**Figure 2:**
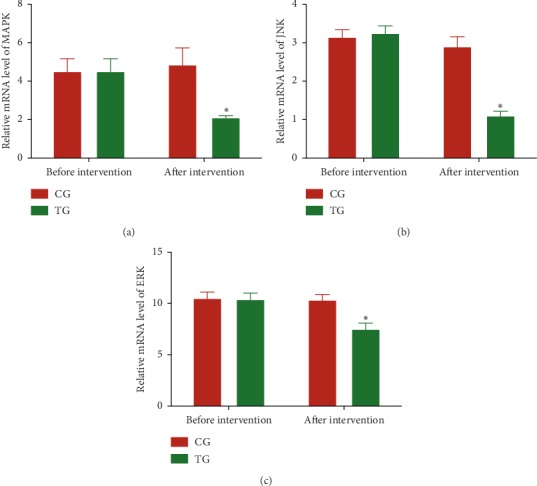
Relative mRNA levels of AMPK, JNK, and ERK in the peripheral blood leukocytes (PBLs) of the patients with coronary heart disease. (a) AMPK, Kelch-like ECH-associated protein. (b) JNK, c-JUN N-terminal kinase. (c) ERK, extracellular signal-regulated kinase. TG, Tai Chi intervention group (*n* = 18); CG, common-care group (*n* = 12). The statistical difference was significant if *P* < 0.05.

Before the intervention, the statistical difference for relative mRNA levels of MAPK (Figure 2(a)), JNK (Figure 2(b)), and ERK (Figure 2(c)) was insignificant between the two groups (*P* > 0.05). After the 3-month intervention, relative mRNA levels of MAPK (Figure 2(a)), JNK (Figure 2(b)), and ERK (Figure 2(c)) in the TG group were lower than those in the CG group (*P* < 0.05). The results suggest that Tai Chi intervention reduces relative mRNA levels of MAPK, JNK, and ERK levels in the CHD patients.

### 3.15. Tai Chi Reduced Relative Protein Levels of MAPK, JNK, and ERK in the CHD Patients

**Figure 3:**
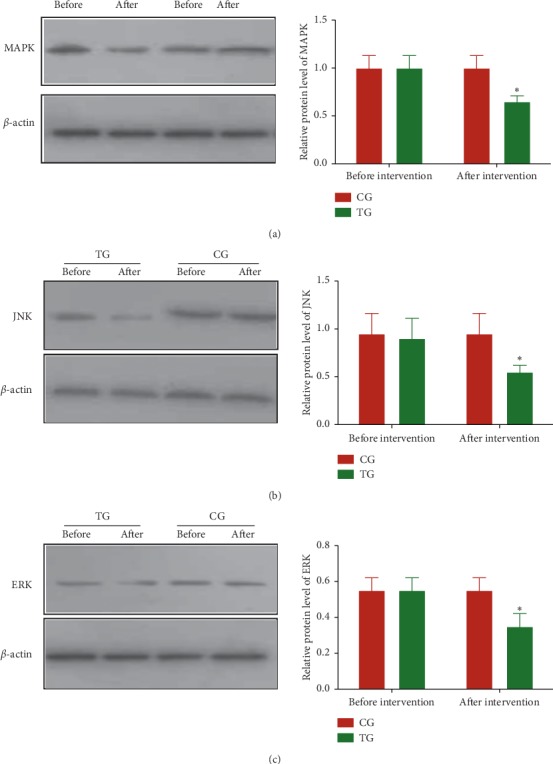
Relative protein levels of AMPK, JNK, and ERK in the peripheral blood leukocytes (PBLs) of the patients with coronary heart disease. (a) AMPK, Kelch-like ECH-associated protein. (b) JNK, c-JUN N-terminal kinase. (c) ERK, extracellular signal-regulated kinase. TG, Tai Chi intervention group (*n* = 18); CG, common-care group (*n* = 12). The statistical difference was significant if *P* < 0.05.

Before the intervention, the statistical difference for relative protein levels of MAPK (Figure 3(a)), JNK (Figure 3(b)), and ERK (Figure 3(c)) was insignificant between the two groups (*P* > 0.05). After the 3-month intervention, relative protein levels of MAPK (Figure 3(a)), JNK (Figure 3(b)), and ERK (Figure 3(c)) in the TG group were lower than those in the CG group (*P* < 0.05). The results suggest that Tai Chi intervention reduces the relative protein levels of MAPK, JNK, and ERK levels in the CHD patients.

### 3.16. MiR-126 Regulated Relative Protein Levels of MAPK, JNK, and ERK

**Figure 4:**
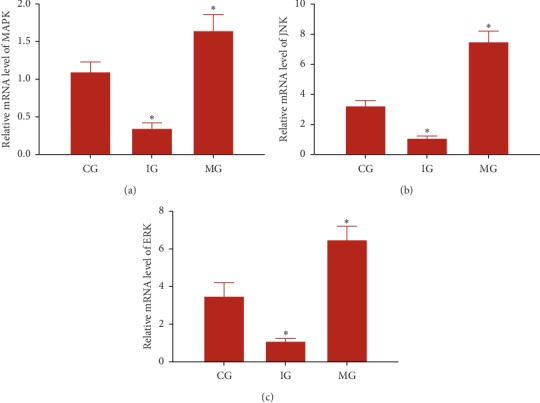
The effects of miR-126 on relative mRNA levels of MAPK pathway-associated molecules. (a) AMPK, Kelch-like ECH-associated protein. (b) JNK, c-JUN N-terminal kinase. (c) ERK, extracellular signal-regulated kinase. CG, control group; IG, miR-126 silence group; MG, miR-126 mimic group (*n* = 8 for each group). The statistical difference was significant if *P* < 0.05.

After miR-126 mimic infection, relative protein levels of MAPK (Figure 4(a)), JNK (Figure 4(b)), and ERK (Figure 4(c)) were increased when compared with the CG group (*P* > 0.05). In contrast, after miR-126 shRNA infection, relative protein levels of MAPK (Figure 4(a)), JNK (Figure 4(b)), and ERK (Figure 4(c)) were reduced when compared with the CG group (*P* < 0.05). The results suggest that miR-126 regulates relative protein levels of MAPK, JNK, and ERK levels.

### 3.17. MiR-126 Regulated Relative Protein Levels of MAPK, JNK, and ERK

**Figure 5:**
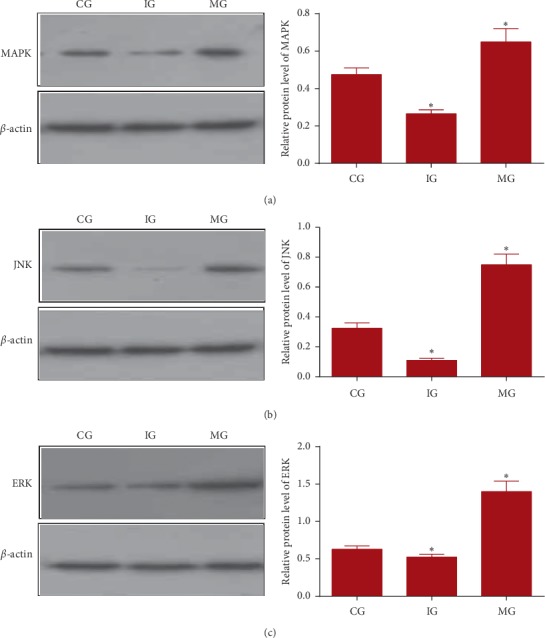
The effects of miR-126 on relative protein levels of MAPK pathway-associated molecules. (a) AMPK, Kelch-like ECH-associated protein. (b) JNK, c-JUN N-terminal kinase. (c) ERK, extracellular signal-regulated kinase. CG, control group; IG, miR-126 silence group; MG, miR-126 mimic group (*n* = 8 for each group). The statistical difference was significant if *P* < 0.05.

After miR-126 mimic infection, relative protein levels of MAPK (Figure 5(a)), JNK (Figure 5(b)), and ERK (Figure 5(c)) were increased when compared with the CG group (*P* > 0.05). In contrast, after miR-126 shRNA infection, relative protein levels of MAPK (Figure 5(a)), JNK (Figure 5(b)), and ERK (Figure 5(c)) were reduced when compared with the CG group (*P* < 0.05). The results suggest that miR-126 regulates relative protein levels of MAPK, JNK, and ERK levels.

### 3.18. MiR-126 Had a Strong Relationship with the Values of EATV and HR and QoL Scores

**Figure 6:**
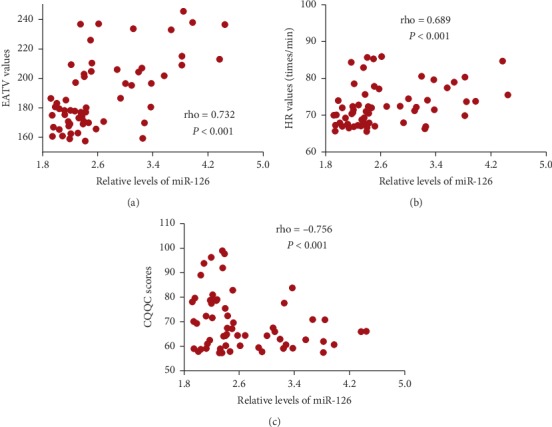
Pearson's correlation coefficient analysis of the relationship between relative levels of miR-126 and the values of EATV and HR or CQQC scores. (a) The relationship between relative levels of miR-126 and the values of EATV. (b) The relationship between relative levels of miR-126 and the values of HR. (c) The relationship between relative levels of miR-126 and the values of CQQC scores. There is a strong positive relation if rho value falls within 0.5 and 1. There is a strong negative relation if rho value falls within −0.5 and −1.

Pearson's correlation coefficient test showed that the increase in the levels of miR-126 would result in the increase in the values of EATV (Figure 6(a)) and HR (Figure 6(b)) and the decrease in the CQQC scores (Figure 6(c), *P* < 0.05). The results suggest that the relative levels of MiR-126 have a strong positive relationship with the values of EATV and HR and a strong negative relationship with QoL scores.

## 4. Discussion

This study validated the efficacy of Tai Chi in patients with CHD after PCI by randomized controlled trials. Intervention studies in the CHD patients showed that Tai Chi could help reduce the EATV, HR, BP, and fat percent and improve CQQC scores. This was consistent with the assumptions presented in this study. EATV is an independent indicator of long-term main adverse cardiovascular events in CHD patients after PCI and is affected by adipose tissue dysfunction. HR variability is also influenced by epicardial fat thickness. QoL assessment is an important gauge of health after PCI. Tai Chi has the protective function for the patients after PCI by affecting these parameters and improving adipose tissue function.

High-level HR is an important predictor of cardiovascular events and all-cause mortality in patients with CHD. The acceleration of HR is not only the performance of CHD and myocardial infarction but also an important cause of cardiovascular disease [48]. Lower HR values are beneficial for infarction and surrounding area angiogenesis and establishment of collateral circulation, restoring cardiac function and improving prognosis. The results of this study showed that Tai Chi reduced HR, and the mean HR of the TG group decreased by about 5 beats/min, while the CG group increased by about 5 beats/min, and the difference between the two was about 10 beats/min. When HR was reduced by 10 beats/min or more, the incidence of heart failure was significantly decreased in patients with myocardial infarction, and the peak of cardiac ejection fraction was significantly increased. For every 10 beats/min increase in HR in hypertensive patients, the risk of major cardiovascular events increased by 8% [49]. Tai Chi reduced HR and improved coronary perfusion to reduce the occurrence of cardiac complications and accidents.

The results of this study showed that SBP and DBP decreased in the TG group, which is consistent with the results of Modesti [50]. The possible reason is that the slowing of the respiratory rate can increase the baroreceptor reflex, reduce the chemoreceptor activity, increase the baroreflex sensitivity, and decrease BP. DBP responds to peripheral blood vessel resistance and is controlled by sympathetic nerve activity. Slow breathing can stimulate the BlackBerry reflex through the change of tidal volume, increase the inhibition of nerve impulse and duration, reduce the sympathetic nerve excitability of skeletal muscle, decrease the peripheral vascular resistance, and finally reduce DBP [50].

Myocardial oxygen consumption is mainly affected by ventricular wall tension, myocardial contractility, and heart rate. The tension of the ventricular wall is proportional to the SBP. Therefore, HR ∗ SBP, that is, RPP, can be used as an index for estimating myocardial oxygen consumption. RPP >12000 mmHg/min indicates an increase in myocardial oxygen consumption [51]. A decrease in RPP indicates a decrease in myocardial oxygen consumption and cardiac load, an increase in effective circulation, and an improvement in cardiac function. The results in this study showed that after Tai Chi, the patient's RPP decreased, and the number of patients with RPP >12000 mmHg/min in the TG group was significantly reduced, suggesting that Tai Chi can help reduce myocardial oxygen consumption.

Quality of life (QoL) has become an important factor in evaluating treatment outcomes. The decline of HR, BP, and RPP improved coronary blood supply, reduced oxygen consumption, and relieved the symptoms such as chest tightness and heart-capture. However, the specific mechanism of Tai Chi on the QoL effect of patients with CHD after PCI is still to be further verified.

Obesity can be divided into two types according to the location of fat distribution: one is centripetal obesity and the other is peripheral obesity. Studies have shown that abdominal obesity, especially excessive visceral fat, is the leading cause of diabetes [52], cardiovascular [53], and cerebrovascular diseases [54]. In abdominal obesity, excessive accumulation of visceral fat has more serious harm to human health. Most obese people are associated with abdominal obesity, and effective reduction of abdominal obesity is more conducive to health. In this experiment, the changes of abdominal subcutaneous fat area and visceral fat area were compared after the exercise intervention. At the same time, to accurately reflect the changes of abdominal fat, CT tomography was used to measure the subcutaneous fat area and visceral fat. Meanwhile, dual-energy X-rays were used for measuring the fat mass of the abdominal android region and the hip gynoid region. The present findings demonstrated that Tai Chi effectively reduced the amount of abdominal fat in CHD patients after PCI.

Tai Chi is effective for weight loss and is recommended as the first-line treatment for obesity [55, 56]. Although the effects of Tai Chi on fat metabolism have not been reported, an animal test showed that aerobic exercise increased acyl-CoA oxidase 1 and monoglyceride lipase expression in adipose tissue and significantly decreased abdominal fat mass [57]. Tai Chi belongs to a kind of aerobic exercise and should have similar function in regulating fat metabolism. MAPK pathway plays an important role in the pathogenesis of CHD [31] and is regulated by miR-126 [34]. The present findings demonstrated that Tai Chi intervention reduced the relative levels of miR-126 (Figure 1) and the levels of MAPK signaling (Figures 2 and 3). The levels of MAPK signaling associated molecules were regulated by miR-126 (Figure). MiR-126 has a strong relationship with the values of EATV and HR and QoL scores (Figure 6). Thus, Tai Chi exercises improved CHD risk by affecting MAPK signaling pathway via serum miR-126.

There were some limitations in the present study. This study was a small sample, single-center study, and the present results may have some bias. To control the bias, the subjects in the present study were all from the same department of the same hospital. The disease type was single and the sample size was limited. Whether Tai Chi has universal applicability remains to be further studied. The indicators after the patient was discharged from the hospital were not studied for a long term, such as more than 3 months, so the long-term effect of Tai Chi exercise is still unclear. At baseline, there was no significant difference in HR, BP, RPP, and CQQC scores between the two groups, but the BP, RPP, and CQQC scores of the TG group were slightly better than those of the CG group. The sample size and study population should be increased and the intervention time should be prolonged to explore the impact of long-term Tai Chi on the rehabilitation of PCI patients.

This study compared the effects of Tai Chi on the abdominal fat area of patients. The results of the study showed that Tai Chi had a significant weight loss effect and effectively reduced the visceral fat area. However, this experiment only compared the effects of the exercise intervention on overweight patients and the effects of Tai Chi on the fat contents of the patients with normal weight remained unclear. This experiment mainly compared the differences between the two groups and no mechanism was explored. As we all know, Tai Chi combined with aerobic exercise is the best way to reduce fat, so how to combine resistance training and Tai Chi training is worth further exploring.

## 5. Conclusions

Tai Chi intervention reduced EATV, HR, SBP, RPP, and fat percentage and increased QoL scores in the CHD patients after PCI, so Tai Chi should be developed as an assistant therapy for CHD patients after PCI. After the 3-month intervention, short-term high-intensity Tai Chi training has shorter exercise time and better weight loss than the CG group. It can effectively reduce the body fat and fat% of the patient, especially for reducing the amount of abdominal fat. Tai Chi improves CHD risk by inactivating MAPK/ERK pathway via serum miR-126. It provided a new choice for CHD therapy in patients with adipose tissue dysfunction.

## Figures and Tables

**Table 1:** Baseline characteristics between the two groups.

Parameters	TG	CG	*t* or *χ*^2^ values	*P* values
Age, yr	62.21 ± 7.76	59.65 ± 8.42	0.950^a^	0.349
Gender (male)	18 (94.74)	16 (94.12)	NA	1.000
Average physical activates during a day, hours				
Supine position	8.5 ± 2.7	8.1 ± 2.5	0.689	0.237
Sitting position	8.8 ± 2.4	9.2 ± 2.8	0.564	0.328
Standing position	4.5 ± 1.3	4.2 ± 1.6	0.139	0.741
Walking duration	2.9 ± 1.2	3.1 ± 1.4	0.065	0.846
Occupation	15 (78.95)	10 (58.82)	NA	0.281
Education level, junior high school and below	7 (36.84)	8 (47.06)		
High school, college	9 (47.37)	6 (35.29)	0.661^b^	0.904
University and above	3 (15.79)	3 (17.65)		
Single (yes)	0 (0.00)	2 (11.76)	NA	0.216
Changchun medical insurance	16 (84.21)	14 (82.35)	NA	1.000
Low-salt and low-fat diet	11 (57.89)	8 (47.06)	NA	0.739
Sleep well	16 (84.21)	15 (88.24)	NA	1.000
Smoking	14 (73.68)	13 (76.47)	NA	1.000
Drinking	15 (78.95)	14 (82.35)	NA	1.000
Personal characters				
Character	6 (31.58)	8 (47.06)		
Introvert	8 (42.11)	7 (41.18)	1.500^b^	0.515
Intermediate	5 (26.32)	2 (11.76)		
Health guidance	5 (26.32)	3 (17.65)	NA	0.695
Medical cost (% of salary)				
<20%	2 (10.53)	5 (29.41)		
20–40%	9 (47.37)	7 (41.18)	2.054^b^	0.423
>40	8 (42.11)	5 (29.41)		
Less than one-year PCI	13 (68.42)	12 (70.59)	NA	1.000
The number of disease vessels				
1	7 (36.84)	9 (52.94)		
2	5 ( (26.32)	4 (23.53)	1.108^b^	0.638
3	7 (36.84)	4 (23.53)		
The number of stents				
1	8 (42.11)	8 (47.06)		
2	5 (26.32)	4 (23.53)	0.199^b^	1.000
3	6 (31.58)	5 (29.41)		
Family history				
High blood pressure	11 (57.89)	14 (82.35)	NA	0.156
Diabetes	7 (36.84)	5 (29.41)	NA	0.732
Cardiopulmonary disease	5 (26.32)	8 (47.06)	NA	0.299
Physical activity				
Low	4 (21.05)	3 (17.65)	0.232	0.890
Medium	12 (63.16)	12 (70.59)		
High	3 (15.79)	2 (11.76)		
Daily diet intake				
Energy intake (kcal/d)	2539 ± 49	2418 ± 40	0.129	0.685
Carbohydrate (% of E)	53.6 ± 6.5	57.9 ± 7.2	0.682	0.127
Fat (% of E)	31.9 ± 4.0	29.85 ± 3.4	0.514	0.471
Protein (% of E)	12.1 ± 2.1	12.8 ± 1.9	0.218	0.584
Protein (g/kg BW)	0.81 ± 0.11	0.88 ± 0.13	0.670	0.185
Saturated fat (% of E)	9.6 ± 1.5	9.9 ± 1.75	0.104	0.715
Monounsaturated fat (% of E)	11.1 ± 1.2	10.8 ± 0.9	0.089	0.824
Poly-unsaturated fat (% of E)	7.3 ± 1.2	6.8 ± 1.1	0.241	0.486
Total fiber (g/d)	45.3 ± 6.4	43.2 ± 6.0	0.187	0.819
Medical treatment, cases (%)				
Danshen	7 (36.84)	6 (35.29)	0.009	0.923
Suxiao Jiuxin	12 (63.16)	11 (64.71)		
BMI, kg/m^2^	25.20 ± 2.17	25.41 ± 2.61	−0.207^a^	0.838
Triglyceride, mM	1.48 ± 0.94	1.31 ± 0.58	0.557^a^	0.582
HDL, mM	1.02 ± 0.23	0.91 ± 0.17	1.286^a^	0.211
LDL, mM	2.07 ± 0.78	2.27 ± 0.90	−0.607^a^	0.550
Total cholesterol, mM	3.73 ± 0.93	6.32 ± 9.41	−1.028^a^	0.314
Creatinine, mM	79.67 ± 32.87	77.54 ± 25.13	0.190^a^	0.851
Fasting blood glucose, mM	6.00 ± 1.20	6.28 ± 2.57	−0.343^a^	0.735
P Receptor blocker, nM	37.24 ± 33.43	42.02 ± 33.50	−0.428^a^	0.671

*Note.* a indicates independent sample *t*-test; b indicates Fisher's exact probability test. The statistical difference was significant if *P* < 0.05.

**Table 2:** The effects of Tai Chi on EATV (cm^3^).

RPP	TG	CG	t values	*P* values
As-treated analysis, cases	*n* = 18	*n* = 12		
Before intervention	216.54 ± 25.78	211.38 ± 31.15	0.524	0.451
3-month intervention	184.05 ± 27.15	204.37 ± 35.13	1.790	0.019^*∗*^
ITT analysis	*n* = 19	*n* = 17		
Before intervention	213.45 ± 19.18	209.31 ± 16.07	0.117	0.765
3-month intervention	172.36 ± 22.19	201.14 ± 17.26	1.956	0.008^*∗*^

*Note.* ^*∗*^ *P* < 0.05 versus the CG group.

**Table 3:** As-treated analysis of the effects of Tai Chi on HR and BP.

Parameters	TG, *n* = 18	CG, *n* = 12	*t* values	*P* values
Before intervention				
HR, times/min	77.39 ± 6.37	76.33 ± 7.57	0.412	0.683
SBP, mmHg	128.00 ± 18.52	132.75 ± 16.10	−0.724	0.475
DBP, mmHg	70.22 ± 12.00	74.75 ± 12.06	−1.010	0.321
After intervention				
HR, times/min	72.83 ± 7.12	81.50 ± 8.03	−3.106	0.004^*∗∗*^
SBP, mmHg	126.28 ± 11.97	136.67 ± 12.68	−2.275	0.031^*∗*^
DBP, mmHg	69.67 ± 13.16	79.33 ± 12.01	−2.039	0.051

*Note.* ^*∗*^ *P* < 0.05 versus the CG group and ^*∗∗*^*P* < 0.01 versus the CG group.

**Table 4:** ITT analysis of the effects of Tai Chi on HR and BP.

Parameters	TG, *n* = 19	CG, *n* = 17	*t* values	*P* values
Before intervention				
HR, times/min	76.74 ± 6.81	77.06 ± 8.22	−0.128	0.899
SBP, mmHg	127.95 ± 18.00	136.29 ± 16.69	−1.437	0.160
DBP, mmHg	71.1 ± 12.28	76.71 ± 12.00	−1.381	0.176
After intervention				
HR, times/min	72.42 ± 7.14	80.71 ± 8.53	−3.171	0.003^*∗∗*^
SBP, mmHg	126.32 ± 11.63	139.06 ± 13.91	−2.933	0.005^*∗∗*^
DBP, mmHg	70.58 ± 13.40	79.94 ± 11.59	−2.229	0.032^*∗*^

*Note.* ^*∗*^ *P* < 0.05 versus the CG group and ^*∗∗*^*P* < 0.01 versus the CG group.

**Table 5:** The effects of Tai Chi on Rate-Pressure-Product (RPP, mmHg/min).

RPP	TG	CG	*t* values	*P* values
As-treated analysis, cases	*n* = 18	*n* = 12		
Before intervention	9879.94 ± 1485.18	10200.33 ± 1391.51	−0.593	0.558
3-month intervention	9182.00 ± 1118.60	11178.92 ± 1775.86	−3.790	0.001^*∗∗*^
ITT analysis	*n* = 19	*n* = 17		
Before intervention	9794.42 ± 1490.70	10581.88 ± 1840.03	−1.417	0.165
3-month intervention	9133.21 ± 1107.69	11272.65 ± 1968.21	−3.956	0.001^*∗∗*^

*Note.* ^*∗∗*^ *P* < 0.01 versus the CG group.

**Table 6:** The effects of Tai Chi on CQQC scores.

RPP	TG	CG	*t* values	*P* values
As-treated analysis	*n* = 18	*n* = 12		
Before intervention	73.72 ± 17.30	66.67 ± 10.10	1.407	0.170
3-month intervention	87.61 ± 12.75	70.33 ± 14.13	3.483	0.002^*∗∗*^
ITT analysis	*n* = 19	*n* = 17		
Before intervention	73.79 ± 16.81	69.88±1L52	0.820	0.418
3-month intervention	86.95 ± 12.73	72.47 ± 13.60	3.299	0.002^*∗∗*^

*Note.* ^*∗∗*^ *P* < 0.01 versus the CG group.

**Table 7:** Changes in dietary calories and physical activity before and after intervention in each group in the as-treated analysis.

Parameters	TG		CG	
	Before	After	Before	After
Dietary calories (Kcal·d^−1^)	1284 ± 366	1273 ± 359	1365 ± 419	1358 ± 417
Physical activity (METs- hr. wk^−1^)	512 ± 239	522 ± 248	529 ± 246	534 ± 151

**Table 8:** Fat changes before and after intervention in the as-treated analysis (kg).

Parameters	TG		Changes, mean (95% CI)	CG		Changes, mean (95% CI)
	Before	After		Before	After	
Weight	67.3 ± 6.1	62.0 ± 6.7^*∗*^	−4.3 (−5.6,−1.9)	66.7 ± 6.8	64.9 ± 6.2	−1.7 (−2.8, −0.6)
Fat%	38.14 ± 2.3	34.62 ± 1.9^*∗*^	−3.5 (−4.6, −1.5)	38.4 ± 2.3	36.3 ± 2.1	−2.0 (−2.8, −1.4)
Fat weight	25.9 ± 3.2	22.85 ± 3.0^*∗*^	−2.9 (−4.0, −1.7)	25.7 ± 3.5	23.7 ± 3.3	−2.0 (−2.7, −1.3)
Nonfat weight	39.6 ± 3.7	39.1 ± 2.9	−0.5 (−1.0, 0.1)	39.0 ± 3.5	41.2 ± 7.7	2.2 (−2.0, 6.3)
Right leg fat	5.9 ± 0.5	4.5 ± 0.5^*∗*^	−0.4 (−0.6, −0.2)	5.0 ± 0.8	4.7 ± 0.9	−0.3 (−0.5, −0.1)
Left leg fat	4.9 ± 0.5	4.4 ± 0.6^*∗*^	−0.5 (−0.7,−0.3)	4.91 ± 0.8	4.85 ± 0.8	−0.3 (−0.5,−0.2)
Right arm fat	1.6 ± 0.3	1.4 ± 0.2^*∗*^	−0.2 (−0.3,−0.1)	1.6 ± 0.3	1.5 ± 0.2	−0.1 (−0.2, 0.0)
Left arm fat	1.6 ± 0.3	1.3 ± 0.2^*∗*^	−0.2 (−0.4, −0.1)	1.6 ± 0.2	1.5 ± 0.3	−0.1 (−0.2, 0.0)
Body fat	11.7 ± 2.1	10.2 ± 2.1^*∗*^	−1.6 (−2.2, −0.9)	11.5 ± 2.2	10.8 ± 2.0	−1.2 (−1.6, −0.7)
Head fat	1.1 ± 0.7	1.1 ± 0.8	0.0 (−0.01, 0.05)	1.2 ± 0.1	1.2 ± 0.1	0.01 (−0.02, 0.03)
Belly (android) fat	2.0 ± 0.4	1.7 ± 0.4^*∗*^	−0.3 (−0.4, −0.2)	2.0 ± 0.4	1.9 ± 0.4	−0.2 (−0.3, −0.1)
Hip (gynoid) fat	4.6 ± 0.5	4.1 ± 0.6^*∗*^	−0.5 (−0.7, −0.4)	4.6 ± 0.6	4.2 ± 0.6^*∗*^	−0.4 (−0.6, −0.3)
Visceral fat (cm^2^)	68.9 ± 24.7	59.9 ± 19.1^*∗*^	−9.08 (−17.8, −0.4)	69.3 ± 23.4	64.9 ± 21.9	−6.31 (−11.4, −1.2)
Subcutaneous fat (cm^2^)	248.4 ± 61.2	213.4 ± 51.0^*∗*^	−35.1 (−57.4, −12.7)	244.9 ± 62.3	227.5 ± 51.8	−17.4 (−33.8, −0.9)

*Note.* ^*∗*^ *P* < 0.05 versus before intervention.

**Table 9:** Balance performance changes before and after intervention.

Parameters	TG		Changes, mean (95% CI)	CG		Changes, mean (95% CI)
	Before	After		Before	After	
Eyes open	65.3% (59.1%−76.4)	82.5% (74.3%–88.5%) ^*∗*^	17.2% (6.9%, 28.4%)	67.9% (57.6%−78.1)	71.9% (64.7%–79.8%)	3.1% (−0.8%, 7.5%)
Eyes closed	57.9% (49.2%−70.4)	74.5% (69.2%–86.2%) ^*∗*^	16.6% (6.4%, 29.7%)	58.2% (50.4%−69.5)	62.7% (55.2%–73.1%)	3.4% (−1.2%, 8.9%)

*Note.* ^*∗*^ *P* < 0.05 versus before intervention.

## Data Availability

The data for the current study are available from the corresponding author upon reasonable request.
